# Integrating stakeholder perspectives in the co-design of medical device software to monitor Parkinson’s disease symptoms- A case-study

**DOI:** 10.1016/j.mex.2026.103935

**Published:** 2026-05-07

**Authors:** Rituja A. Raut, Barbara Polus, Quoc Cuong Ngo, Nemuel D. Pah, Robyn Clay-Williams, Dinesh K. Kumar

**Affiliations:** a RMIT University, School of Engineering, Melbourne, 3000, Victoria, Australia; b Macquarie University, Faculty of Medicine, Health and Human Sciences, Sydney, 2109, New South Wales, Australia

**Keywords:** Co-design, Software as a medical device (SaMD), Stakeholder engagement, Parkinson’s disease, Digital health

## Abstract

There has been a rapid proliferation of AI-based medical devices and Software as a Medical Device (SaMD), largely due to their ease of deployment and scalability. Despite these advantages, many such technologies face limited clinical acceptance and reduced effectiveness in real-world healthcare settings. This gap is frequently attributed to technology-driven development approaches that insufficiently involve end users and other key stakeholders. This paper describes a structured co-design approach applied to the development of NCare, a smartphone-based SaMD designed to monitor motor symptoms associated with Parkinson’s disease. Co-design engaged people living with Parkinson’s Disease, clinicians, developers, and commercial stakeholders in iterative system refinement. Workshops, surveys, observational methods, and a trial phase were used to capture experiential, clinical, and contextual insights across different stages of development. This approach enabled the identification of usability challenges, workflow integration requirements, and reporting requirements absent from developer-led approaches. The paper presents a case study of co-design protocol intended to support the development of needs-driven, clinically relevant, and adoptable digital health technologies.

**Method Overview:**

Engage multiple stakeholder groups across stages of device development.

Use iterative co-design activities to elicit experiential and practice-based insights.

Apply feedback-driven refinement and trial deployment to assess real-world usability and implemen-tation considerations.

## Graphical abstract

**Figure:**
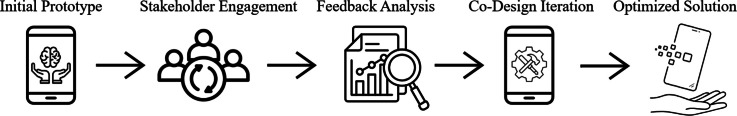

## Specifications table

**Table:** 

Category	Details
Subject area	Medicine and Dentistry
More specific subject area	Parkinson’s Disease
Name of method	Co-design approach for development of a software as a Medical device
Name and reference of original method	Not Applicable
Resource availability	For access to the data, please contact bar-bara.polus@rmit.edu.au

## Background

In an environment characterized by rapid technological advancement and an increasing number of prod-ucts across the medical devices market segments, a successful product is one that effectively meets the needs of all stakeholders while remaining intuitive and accessible to use. Achieving this alignment requires the active participation of all stakeholders throughout the development process [1]. Co-design is a collabora-tive approach in which stakeholders work together to develop or optimize solutions through design-thinking processes [2]. Its origins can be traced to Participatory Design movements in Scandinavia during the 1970s, where workers in Norway and Sweden were directly involved in shaping workplace technologies to improve productivity and working conditions [3]. These principles have increasingly informed healthcare innovation as medical technologies have grown in complexity and impact. Historically, medical device development has been dominated by a “technology push” model, prioritizing technical performance over usability and experiential integration. This engineering-centered paradigm frequently overlooked the practical realities of use, resulting in devices that met regulatory or technical benchmarks but failed to address patient needs meaningfully [4]. As patients and clinicians are the primary end users of medical devices, their involve-ment from concept to delivery is essential. The World Health Organization has highlighted that many medical devices fail due to limited contextual understanding and insufficient user engagement, reinforcing the importance of participatory approaches in healthcare technology development [5,6]. While medical professionals possess deep clinical expertise, they typically lack the specialized technical skills required to directly assist in the device design, software programming, AI models or signal processing aspects of the technology. However, clinicians can reveal subtle, "latent" needs that are invisible to an engineer. For example, a recent study showed that adding haptic feedback to robotic surgery systems can significantly re-duce clinician workload. This finding emerged from trials conducted with practicing clinicians, highlighting the importance of real-world clinical involvement to addresses the correct clinical problems [7]. Financial sustainability is another critical consideration in medical device development. High capital investment and production costs necessitate early assessment of commercial viability. Commercialization experts play an important role in identifying market opportunities and reimbursement pathways, without which publicly funded innovations may struggle to transition into healthcare systems. A review of fifty-five healthcare co-design studies published between 2016 and 2024 reported a sharp increase in publications in 2023, re-flecting growing interest in co-design. However, substantial inconsistencies were identified: 44% of studies lacked an explicit definition, frameworks varied from simplified three-stage to more comprehensive multi-step models, and stakeholder roles ranged from informants to co-designers [8]. Some definitions focused solely on end-user involvement [9], highlighting the need for clearer, scalable co-design guidance [8]. Co-design is increasingly recognized by journals, funding bodies such as the Medical Research Future Fund, and regulatory agencies including the Therapeutic Goods Administration and the Medicines and Healthcare products Regulatory Agency [10,11]. Despite this recognition, co-design practice remains inconsistent and insufficiently rigorous, lacking validated protocols for systematic and measurable implementation. This pa-per is a case-study which addresses this gap by developing and examining a structured co-design protocol for healthcare technology development.

## Method details

### Development history

The concept for this application originated in 2016, sparked by an informal conversation between a student and a clinician. Their discussion centered on the potential for a digital platform to streamline how providers monitor patient symptoms remotely. Early engagement with clinical stakeholders facilitated the identification of core requirements from an end-user perspective. This collaborative process transitioned into a structured brainstorming phase, aimed at translating clinical needs into functional technical specifi-cations. A prototype for application "NCare", a software as a medical device (SaMD) AI tool that assesses patients for three motor symptoms associated with Parkinson’s disease was developed. NCare incorporates three AI models: HandDIAG, VoiceDIAG and VidDIAG.

HandDIAG is an SaMD to identify changes in micrographia and acceleration / force of handwriting. VoiceDIAG estimates the hoarseness of voice. VidDIAG helps in identifying hypomimia of facial features.

### Early stage device development

This study investigated the use of co-design to optimize NCare. The first co-design and consumer en-gagement step was conducted about 8 months prior to this protocol. After obtaining ethics approval from the RMIT Human Research Ethics Committee (Project ID 27,000), members of the research team demonstrated the HandDIAG, VoiceDIAG and VidDIAG Apps at the annual Parkinson’s Australia conference (2025), and invited volunteers to use the device. 54 people volunteered and used the devices and provided feed-back. Feedback was also taken from eight clinicians who attended the conference. Based on the feedback, number of changes were made such as integration of HandDIAG, VoiceDIAG and VidDIAG into a single application, i.e. NCare. It was also acknowledged that the device needed to be suitable for home use by people living with PD (PLwPD) rather than being only for in-clinic use. Another acknowledgment at the end of the conference was that the co-design protocol was too informal.

### Key stakeholders

The co-design process engaged four stakeholder groups selected to represent perspectives relevant to Software as a Medical Device (SaMD) development:

• **People living with Parkinson’s disease (PLwPD):** They were engaged as end-users throughout the in-person co-design workshops and structured surveys, providing real-world insights grounded in lived experience. Their involvement ensured that the application was developed in alignment with user requirements, with particular emphasis on usability and accessibility. Through facilitated discus-sions and survey participation, participants highlighted practical needs related to interface simplicity, accessibility features, symptom-tracking relevance, and everyday use within their care routines. These insights directly informed iterative refinements to the application’s design, functionality, and feature configuration, ensuring that development decisions were responsive to real-world user expectations rather than solely technology-driven assumptions. Their input was incorporated into successive re-finement cycles, ensuring that usability and accessibility considerations remained central throughout development.
• **Clinicians:** Clinicians participated as clinical domain experts through structured surveys and tar-geted feedback review. Their input provided real-world insights into diagnostic practice, assessment validity, report format requirements, and integration within routine clinical workflows. Clinicians evaluated the clinical relevance of assessment tasks, interpretability of generated reports, and align-ment with established severity scales. Their feedback informed refinements to assessment parameters, report structure, and clinical positioning of the application as a consultation-support and disease-progression monitoring tool rather than a standalone diagnostic replacement. These contributions ensured that development decisions were grounded in clinical practicality and workflow feasibility.
• **Application Developers:** Application developers were involved throughout all phases as technical implementers, translating stakeholder feedback into feasible system modifications while ensuring functional integrity and compliance with technical constraints.
• **Commercial advisors:** Commercial considerations were incorporated to assess adoption feasibility and scalability within healthcare systems. This informed strategic positioning and long-term sustain-ability planning.

Stakeholder engagement occurred sequentially and iteratively, with feedback from each group informing subsequent refinement cycles. While stakeholder input significantly shaped design evolution, final imple-mentation decisions were guided by considerations of technical feasibility, clinical appropriateness, and regulatory alignment.

### Participant survey

A workshop for people living with Parkinson’s disease (PLwPD) was designed and advertised in the Parkinson’s Australia monthly magazine. Parkinson’s Australia is the peak body representing PLwPD.

Participants who responded to the advertisement were invited for an informal lunch for the co-design meeting. The NCare SaMD was demonstrated to participant, including details of its development to monitor Parkinson’s disease (PD) symptoms and generate reports for clinicians. In line with co-design principles, end users, PLwPD, were engaged as active contributors to the design process, providing experiential input to inform application refinement from an end user perspective. To support this participatory approach, ob-servational data was collected along with survey responses to capture interaction patterns, communication, and collaborative engagement during the session. These observations also informed reflections on how the co-design process and survey facilitation could be improved. Feedback focused on identifying areas for im-provement, assessing the participants’ ability to use the application independently, and exploring anticipated usage patterns once the application was fully developed.

#### Participants

The participants included six potential end users (PLwD), two application developers, and two facilita-tors who participated in an in-person co-design session. The session was intentionally structured to create a shared design space where the stakeholders could engage collaboratively. End users interacted hands-on with the device, shared their experiences, and provided feedback grounded in their lived experience, while developers contributed technical knowledge and design intent. The research team also collectively observed every participant and shared the observations. This reciprocal exchange supported collective understand-ing and knowledge sharing across the participants (PLwPD), application developers, and the facilitator. stakeholder groups.

Facilitators played a central role in sustaining participant engagement throughout the session. They guided the flow of activities, encouraged open discussion, and ensured that participants felt comfortable ex-pressing their views. By fostering an inclusive and supportive environment, facilitators helped reduce bar-riers to participation and enabled end users to provide more detailed, reflective, and constructive feedback. This level of engagement was essential for generating insights that informed refinement of the application and its intended use. In addition, the presence of application developers within the session enabled direct dialogue between end users and those responsible for the design and future development of the application. Participants were able to ask questions, seek clarification, and discuss anticipated future features, contribut-ing to a shared understanding of current functionality and long-term vision. This interaction strengthened stakeholder participation and supported a co-design process grounded in mutual learning rather than one-directional feedback collection.

#### Study design

• **Recruitment and Invitation:** Participants for the co-design workshop were recruited through an open advertisement distributed via neurology clinics and the Parkinson’s Australia newsletter. *Re*-cruitment occurred over approximately two months. Interested individuals voluntarily contacted the research team in response to the advertisement. Seven individuals responded to the advertise-ment. All respondents met the inclusion criteria, and six participants ultimately attended the in-person workshop. One individual was excluded from participation due to interstate geographical con-straints.Eligible participants were subsequently contacted via email and invited to attend an in-person survey session, with scheduling based on their availability. Inclusion criteria were self-reported con-firmed diagnosis of Parkinson’s disease, ability to attend an in-person session and capacity to provide informed consent. Participants were in early-stage Parkinson’s disease (Stage 1). No upper age limit was imposed and there were no exclusion criteria. Assistance with travel to the survey location (RMIT University, Melbourne) was offered to participants residing within the Melbourne metropolitan area to minimize barriers to participation. The final group size of 6 participants was considered appropriate for an interactive co-design workshop, allowing in-depth discussion and active engagement among participants.
• **Session Setting and Duration:** The survey sessions were conducted in person at RMIT University. With informed consent, the session was recorded. All participants provided their written consent for participation and recording prior to commencement. The session lasted approximately two hours and was conducted in a group setting, allowing participants to interact with each other, as well as with facilitators and application developers. Light refreshments were provided during the session to enhance participant comfort and foster a relaxed and inclusive atmosphere. Individuals who feel unwelcome or uneasy in such settings may be less likely to engage fully in the co-design process. Therefore, establishing trust and cultivating positive relationships with participants is essential to supporting meaningful and authentic contributions [12,13]
• **Introduction to the survey:** The session began with an introduction outlining the purpose of the study and emphasizing the importance of engaging all stakeholders in the application development process. To support effective participation, an overview of Parkinson’s disease–related terminology was presented using clear, lay-friendly language, informed by existing literature, to establish a shared understanding of key concepts referenced in the application and survey, consistent with co-design best practice[14].
• **Application Demonstration and Data Collection:** Following the introduction, participants were shown the application interface and guided through the data collection process to familiarize them with its functionality. The application included three assessment tasks handwriting, voice, and facial expression which were designed to capture key symptom indicators. Participants then collected data using the application, with facilitators available to provide assistance when required. Observations indicated that participants were able to complete the tasks without difficulty and found the interface intuitive and easy to use.
• **Survey Administration:** Upon completion of the co-design activities, participants were invited to complete an online survey. The survey was designed to capture demographic and clinical charac-teristics, digital literacy, symptom management practices, usability perceptions, accessibility pref-erences, and attitudes toward data sharing. Demographic and clinical information, including age and self-reported stage of Parkinson’s disease using the Hoehn and Yahr classification was collected to contextualize usability and engagement findings across varying disease severities. Participants’ levels of digital literacy were also assessed to better understand how familiarity with technology in-fluenced interaction with the application. The survey also explored participants’ existing approaches to symptom monitoring and management, including how frequently they reflect on their symptoms and their perceptions of the role digital technologies may play in supporting their care. Usability and design-related questions examined perceived ease of regular application use, preferred additional features (e.g., medication reminders, symptom diaries, exercise guides, and clinician communica-tion), and accessibility preferences such as voice commands and larger interface buttons. Likert-type items assessed comfort with sharing health data through the application and with clinician access to app-generated data. The survey concluded with an open-ended item inviting further suggestions for improvement. The survey incorporated categorical, Likert-type, ranking-style, and open-ended ques-tions to capture both structured responses and experiential feedback. Findings from the survey were synthesized to inform iterative refinement of application functionality and design.

#### Key takeaways

The co-design surveys conducted during this study revealed gaps that warrant consideration in any co-design activity involving stakeholders. These factors have a direct impact on the quality and reliability of the insights generated through surveys. To obtain findings that meaningfully inform improvements to a product or service, co-design activities should be designed to minimize practical, ethical, and environmental barriers to participation. Table 1 summarizes the key observations from the co-design workshop with end users and outlines corresponding co-design implications to address these issues in future.

**Table 1:** Lessons learned from co-design workshops and implications for future practice.

Category	Observations	Co-design implication
Facilitation style	Participants were dependent on facilitators to carry out activities.	Excessive guidance reduces observational validity. Adopt a facilitative, observer-first role and intervene only when required. This will minimize facilitator bias in usability observation
Physical environment	Environmental factors may constrain open expression. Limited privacy and visibility from outside may have affected comfort	Use larger, private spaces to support psychological safety for co-design environments.
Qualitative depth	Open-ended questions in surveys received shallow response.	In-person discussions combined with surveys can yield deeper insights.
Logistics	Food placement on the discussion table was a timely distraction	Session arrangements should reduce distractions while maintaining comfort. Refreshments should be placed separately from the discussion area
Survey design	Errors were identified during survey use	Tools should be tested internally prior to workshop implementation
Contingency planning	A need for printed backups was identified	Technical failures can risk exclusions. Redundant formats like paper-based tools shall be used.
Engagement over time	Repetition of tests may reduce participant engagement	Co-design sessions should maximize participant engagement without task monotony.

In addition to the insights gained from the co-design sessions, several gaps were identified within the survey questions themselves. This highlights the importance of designing survey instruments that include meaningful and actionable questions capable of directly informing application development. Table 2 sum-marizes how the inclusion of additional questions could have provided more targeted insights and further supported the refinement of the application.

**Table 2:** Additional survey dimensions and their relevance to application refinement.

Category	Justification for Inclusion in Survey
Color contrast and visual design	Quantitative evidence indicates that people with Parkinson's disease experience reduced contrast sensitivity across both central and peripheral vision. Including survey questions on color contrast preferences and visibility would have enabled developers to optimize interface color schemes for improved readability and accessibility [15].
Vision-related impairments	Parkinson's disease can cause a range of visual disturbances, including blurred vision, double vision, dry or irritated eyes, and difficulty reading. Surveying vision-related challenges would have informed design adjustments such as font clarity, layout spacing, and visual task presentation [16].
Type of smartphone used (Android or iOS)	Understanding the distribution of Android and iOS users would have guided platform prioritization and ensured that development efforts focused on optimizing the application for the most commonly used operating systems.
Error recovery and task restart	Motor symptoms such as tremor may increase the likelihood of unintended errors during task execution. Survey questions assessing how easily participants could recover from errors or restart tasks would have helped developers design more forgiving and userfriendly error recovery mechanisms.
Touch sensitivity and accidental input	Accidental touches are common due to motor impairments associated with Parkinson's disease. Capturing user feedback on touch sensitivity would have supported the implementation of safeguards to prevent unintended interruptions during test procedures.
Text size and readability	Assessing whether text size and font style were readable would have provided developers with direct guidance on optimizing typography to accommodate visual and motor limitations, thereby improving overall usability.

#### The trial phase

Participants who visited RMIT University were invited for a 4-week trial of the application. They were provided with a ‘fit for purpose mobile phone’ that has the NCare application installed. This version of the application was updated based on feedback received from the participant survey. The experimental device had no sim card and was mostly devoid of other Apps. Using the NCare App did require a Wi-Fi connection. Participants were asked to use the App at least twice per week, at about the same time of the day, after medication, for 4 weeks unsupervised. The App produced an electronic report which was available under the “Reports” section of the NCare App. The report included analyzed parameters of acceleration and force of handwriting; an analysis of level of fluctuations and noise of voice. A Qualtrics survey link was sent to the participant’s email address. A print copy of the survey and a return paid envelope for your convenience was also sent to with the delivery package. This gave the participants an option to either complete the electronic version of the survey or the paper-based copy of the survey. A printed copy of “Participant Information and Consent Form” was also included in the same package that contained the mobile phone and print copy of the survey. If they were unable to provide us with an electronic copy of your signed consent form, we requested them to sign the paper version and use one of the postage-paid return to sender envelopes to return their signed informed consent form to us prior to beginning the trial of using the NCare App. The purpose of providing a print copy of the PICF and survey was to ensure equity. Demographics of the participants can sometime limit their understanding of the use of digital tools. This action recognized that certain participants might need specific arrangements to participate on an equal level [17].

### Clinician survey

Involving diverse stakeholders in the co-design process is a crucial aspect of creating effective and innovative solutions. Engaging multiple parties ensures that the design process incorporates a wide range of perspectives, experiences, and needs. While the participation of end-users is fundamental to ensure usability and relevance, it is equally important to include subject matter experts at subsequent stages. Their specialized knowledge and technical expertise can refine initial ideas, introduce novel insights, and guide the project toward more advanced and innovative outcomes. This collaborative approach not only enhances the overall quality of the product but also increases its potential impact and sustainability.

#### Clinicians

Based on the literature [18,19] clinicians with the following expertise are being invited: Neurologists, Motor-Disorder nurse, and speech-pathologists. General Practitioners and physiotherapists will be invited in the future. To ensure that the feedback is unbiased, clinicians from both, metropolitan and regional areas will be invited for the study. Five neurologists with specialized expertise in Parkinson’s disease, movement disorders, and speech participated in the co-design process. An Associate Professor with 25 years of experience, having dual academic roles and extensive supervisory responsibilities, contributed insights from his clinical and teaching experience. An early career fellowship-trained movement disorders specialist with expertise in advanced Parkinson’s disease management participated. A consultant physician and geriatrician with a focus on neurodegenerative conditions located in regional Victoria, provided his perspectives. A senior neurologist and movement disorder specialist with over 30 years of experience in the public hospital contributed expertise in complex interventions including confounding factors, Parkinsonian, i.e. different disease with symptoms that are similar to Parkinson’s disease. A speech pathologist with over 25 years of experience give a detailed review based on their in-depth knowledge about vocals and how it affects PLwPD. Together, these clinicians brought substantial clinical and academic experience to support a rigorous and contextually grounded co-design process.

#### Study design

Clinician surveys were conducted via online meetings to accommodate their demanding schedules. To support meaningful and informed feedback, a demonstration video of the application was shared, allowing clinicians to review the user interface and understand the range of assessments embedded within the system. This approach enabled them to evaluate not only the clinical reports generated by the application but also the appropriateness and design of the tasks patients are required to perform. The feedback provided by clinicians led to several important refinements to the application.

#### Survey administration

A structured clinician survey comprising 23 questions, including both closed-ended and open-ended items, was administered online to gather detailed feedback on the application. Closed-ended questions were designed to capture quantitative evaluations of usability, sufficiency, and overall satisfaction, while open-ended questions provided clinicians with opportunities to elaborate on their experiences, identify limi-tations, and suggest modifications to better align the application with clinical practice. This mixed-question approach enabled the collection of both measurable usability indicators and nuanced, practice-driven in-sights relevant to system refinement. The survey also explored multiple domains associated with clinical use and adoption of the application. These included clinicians’ experience with Parkinson’s disease care, such as the number of patients managed and the frequency of PD-related consultations, to contextualize feedback within varying levels of clinical exposure. Participants assessed the perceived usefulness of the application, including the value of specific features, overall functionality, and the relevance of generated outputs for clinical interpretation and treatment planning. Clinicians also evaluated the application’s in-terface, clarity of presentation, and visual design, providing input on aspects that may influence efficiency and usability in day-to-day practice. The survey also examined how the application aligns with existing clinical workflows by gathering information on technologies currently used for PD monitoring and man-agement, as well as the perceived reliability of patient-reported and system-generated symptom severity data. Clinicians were invited to reflect on the potential of the application to support patient engagement and adherence to treatment plans, and to consider the feasibility of integrating application outputs into elec-tronic health record systems. Broader considerations related to clinical validation requirements, regulatory approval pathways, and commercial viability were also explored. Finally, clinicians’ willingness to adopt and use the application in routine practice was assessed, allowing their collective feedback to inform design decisions aimed at improving clinical relevance, usability, and long-term implementation potential.

#### Key takeaways

A feedback session was conducted with participating clinicians to gather their initial impressions and identify areas for improvement. In addition to their survey responses, clinicians provided comments on the validity of assessment tasks, clarity of reporting, and how the application could fit within routine clinical workflows. The feedback was reviewed and organised into key themes, linking specific concerns to corre-sponding design refinements and broader implications for Software as a Medical Device (SaMD) develop-ment. Table 3 presents this structured synthesis, demonstrating how clinician input informed modifications to task design, reporting alignment with clinical scales, and the positioning of the application within existing care pathways.

**Table 3:** Clinician feedback and transferable insights for SaMD development.

Category	Clinician Feedback	Analytical interpretation	Transferable Insight for SaMD Development
Writing Assessment	Discrete writing tasks often cancels out resting tremors observed in PLwPD; stylus input produces more reliable results than finger input.	Task structure and input modality directly influence motor signal validity and detection accuracy.	Digital motor assessments must prioritize continuous movement capture and control for hardware variability to ensure measurement reliability.
Facial expression	Smiling is too automatic to provide meaningful diagnostic information.	Automatic facial expressions lack sufficient diagnostic sensitivity; effort-based motor tasks improve discrimination of hypomimia.	Diagnostic tasks in digital assessments should elicit voluntary, effortful motor responses rather than automatic behaviors.
Speech assessment	Limited phoneme set insufficient to capture tongue movement and pitch variation.	Single-parameter speech tasks under-represent the multidimensional nature of Parkinson speech impairment	Multi modal acoustic analysis improves robustness and sensitivity in digital motor speech assessment.
Reporting & clinical scaling	Outputs should align with Unified Parkinson's Disease Rating Scale (UPDRS).	Clinical adoption depends on interpretability within established assessment frameworks.	Digital health outputs must map to recognised clinical frameworks to support practitioner adoption.
Strategic Utility	Application better suited for triage or GP support than specialist replacement.	Adoption depends on integration within existing care pathways rather than clinical disruption.	SaMD uptake improves when supporting clinicians rather than replacing them.

### Commercial partners

Two potential commercial partners with experience in medical devices startups were invited for their comments. They also have experience in the management of intellectual property and regulatory registration.

## Method validation

### Patient survey

The co-design process provided structured insights into how participants interacted with and perceived the application in real-world use. Feedback gathered through surveys and facilitated discussions identified patterns related to usability, accessibility, symptom-tracking behaviors, perceived clinical relevance, and communication with healthcare providers. These insights were systematically organized to distinguish be-tween descriptive feedback and broader analytical implications for Software as a Medical Device (SaMD) development. The findings demonstrated that ease of use, perceived consultation value, flexibility in moni-toring, and accessibility features were key determinants of acceptability and engagement. Table 4 presents this structured synthesis, linking participant feedback to analytical interpretations relevant to digital motor assessment tool development.

**Table 4:** Key findings from participant survey.

Category	Participant feedback	Co-design tion	Analytical Interpretation SaMD Development
Technology familiarity	Majority comfortable with smartphones	Supports adoption of advanced features	Assumptions of low digital literacy in older PLwPD populations should be empirically validated rather than presumed.
Ease of use	Most found the app easy to use	Validates the current design direction	Low cognitive load and intuitive interface design are critical for engagement in motoraffected populations.
Perceived usefulness	High belief in the application's benefit	Indicates strong alignment with participant priorities	Perceived clinical relevance is a key determinant of sustained engagement with digital health tools.
Data sharing	High comfort with sharing data with doctors	Supports co-designed communication between users and practitioners	Trust in clinician-mediated data pathways enhances acceptability of SaMD systems.
Symptom tracking habits	Many participants do not currently track symptoms	Confirms an unmet need addressed by the application	Digital tools can introduce structured monitoring behaviours not routinely embedded in self-management practices.
Preferred tracking frequency	Preferences varied across participants	Necessitates customized and userdriven notification settings	Personalization and flexibility are central to long-term adherence in digital health interventions.
Most valued feature	Communication with doctors	Reinforces the codesign focus on meaningful outcomes	SaMD tools gain perceived value when they enhance consultation preparedness rather than operate in isolation.
Accessibility needs	Requests for larger buttons, trend access, and reminders	Represents concrete design changes driven directly by participant input	Accessibility features are foundational and not optional in digital systems for motor-impaired populations.

### Clinician survey

The involvement of expertise in the co-design process of development of the application helped the team to understand the shortcomings in the existing version. The survey revealed perspectives of clinicians on various aspects of the application. The summary has been given in Table 5.

**Table 5:** Key findings from clinician survey.

Clinician Survey Feedback	Evidence from Survey	Co-Design Interpretation	Analytical Insights
Challenges in managing PLwPD	Open-ended responses highlighted motor fluctuations & early diagnostic difficulty. 100% responses rated that tremor detection is moderately or very useful	Focusing on more advanced handwriting tests to identify motor fluctuations and expanding the application to greater population to identify PD signs as early as possible	The structure of digital assessment tasks directly influences the sensitivity and accuracy of motor symptom detection.
Existing tools for PLwPD	60% selected "other tools"	Opportunity for introducing one tool to diagnose symptoms in three categories.	Consolidating multi-domain assessments into a single platform can improve workflow efficiency and reduce clinical fragmentation.
Symptom tracking	50% rated that symptom tracking is "slightly useful" while 50% rated very/extremely useful	Indicates heterogeneous clinical needs, suggesting the application should offer configurable or optional features to accommodate varying clinician preferences and use cases.	Variability in clinician preferences requires adaptable and configurable digital systems.
Importance of medication logs	Mean usefulness rated as 3.75 out of 5	Co-design-informed inclusion of medication tracking can enhance clinical interpretation during consultations.	Incorporating treatment context enhances interpretation of motor symptom data.
Usefulness of remote patient monitoring feature	75% rated "moderately useful"	Helps to define target user groups rather than positionsing the tool as universally applicable.	Clear positioning within specific care pathways strengthens practical adoption.
Usefulness of VoiceDLAG feature	75% rated "very useful"	Validates the current design direction and supports continued development of voicebased assessment as a clinically meaningful feature.	Multidimensional speech analysis increases robustness of digital motor assessment.
Clinicians prefer institutional payment models	75% favor insurance/hospital coverage	Co-design interpretation identifies insurance companies and hospitals as key stakebolders, informing early commercialization and reimbursement strategies.	Financial alignment with existing healthcare funding models is essential for implementation.
Adoption of application	Requests included free trials, correlation with MDSUPDRS, insarance coverage, and sensitivity to disease severity.	These insights inform a co-designed adoption strategy, emphasizing clinical validation, alignment with established scales, and financial accessibility.	Mapping outputs to extablished clinical frameworks increases professional trust.
Marketing to clinicians should focus on efficiency	Emphasis on disease progression, reduced consultation time, and support for guiding consultations.	Informs a stakeholder-driven marketing strategy aligned with clinical priorities and real-world use contexts.	Digital tools are more likely to be adopted when they support, rather than disrupt, clinical workflows.

## Conclusion

This study focused on the development of a Software as a Medical Device (SaMD) using a structured co-design approach that systematically integrated input from multiple stakeholder groups. The refinement of the application was guided by stakeholder responses to ensure practical usability, contextual relevance, and functional validity. Co-design workshops with people living with Parkinson’s disease (PLwPD) pro-vided experiential insights into accessibility needs, interface clarity, symptom-tracking preferences, and consultation preparation, leading to modifications in task structure, interface design, and reporting features. Clinician feedback contributed to evaluation of diagnostic task validity, reporting alignment with estab-lished clinical frameworks, and feasibility of integration within existing workflows. These inputs supported refinement of assessment tasks, enhancement of reporting formats, and clarification of the application’s role as a consultation support and monitoring tool rather than a diagnostic replacement. This study outlines a structured and reproducible co-design framework that may inform the development and implementation of other SaMD applications across clinical contexts.

### Limitations

This study focused on co-design approach for the development of a SaMD designed specifically for Parkinson’s disease. The stakeholders involved were considered on the basis of particular requirement for this use-case scenario. Other SaMD may require participation of stakeholders that were not part of this study. These stakeholders might vary according to clinical context and intended use. The co-design structure followed in this study is transferrable, but stakeholder composition may need to be adapted for other SaMD use cases.

## Ethics statements

This study follows the Declaration of Helsinki for human participants, which considers respect for the individual, his or her right to self-determination and the right to make informed decisions regarding par-ticipation in research [20]. Human Research Ethics project approval has been obtained from the RMIT.

Human Research Ethics Committee (Project ID 27,000). Written consent is obtained from all participants. This study was approved by the RMIT Human Research Ethics Committee, the National Statement on Eth-ical Conduct in Human Research (NHMRC, 2007-Updated 2018), Australian Code for the Responsible Conduct of Research (NHMRC & Universities Australia, 2018), Statement on Consumer and Community Involvement in Health and Medical Research (NHMRC & CHF, 2016), the principles of Good Clinical Prac-tice, and within the State or Territory the research is being conducted and Australian laws and regulations, including the Public Health Act 2005.

## CrediT author statement

**Rituja A. Raut**: Investigation, Data Analysis, Writing - Original Draft. **Barbara Polus**: Supervision, Formal Analysis, Writing - Review & Editing. **Quoc Cuong Ngo**: Methodology, Software. **Nemuel D. Pah**: Methodology, Software. **Robyn Clay-Williams**: Writing - Review & Editing. **Dinesh K. Kumar**: Conceptualization, Methodology, Writing - Review & Editing, Supervision, Project administration, Funding acquisition.

## Related research article

None.

## Declaration of competing interest

The authors declare that they have no known competing financial interests or personal relationships that could have appeared to influence the work reported in this paper.

## Data Availability

Data will be made available on request.
